# *Bacteroides fragilis* Enterotoxin Upregulates Matrix Metalloproteinase-7 Expression through MAPK and AP-1 Activation in Intestinal Epithelial Cells, Leading to Syndecan-2 Release

**DOI:** 10.3390/ijms222111817

**Published:** 2021-10-30

**Authors:** Jong Ik Jeon, Keun Hwa Lee, Jung Mogg Kim

**Affiliations:** Department of Microbiology and Institute for Rheumatology Research, Hanyang University College of Medicine, Seoul 04763, Korea; siela@hanmail.net

**Keywords:** *Bacteroides fragilis*, enterotoxin, IECs, MMP-7, syndecan-2

## Abstract

*Bacteroides fragilis* enterotoxin (BFT) produced by enterotoxigenic *B. fragilis* (ETBF) causes colonic inflammation. BFT initially contacts intestinal epithelial cells (IECs) and affects the intestinal barrier. Although molecular components of the gut epithelial barrier such as metalloproteinase-7 (MMP-7) and syndecan-2 are known to be associated with inflammation, little has been reported about MMP-7 expression and syndecan-2 shedding in response to ETBF infection. This study explores the role of BFT in MMP-7 induction and syndecan-2 release in IECs. Stimulating IECs with BFT led to the induction of MMP-7 and the activation of transcription factors such as NF-κB and AP-1. MMP-7 upregulation was not affected by NF-κB, but it was related to AP-1 activation. In BFT-exposed IECs, syndecan-2 release was observed in a time- and concentration-dependent manner. MMP-7 suppression was associated with a reduction in syndecan-2 release. In addition, suppression of ERK, one of the mitogen-activated protein kinases (MAPKs), inhibited AP-1 activity and MMP-7 expression. Furthermore, the suppression of AP-1 and ERK activity was related to the attenuation of syndecan-2 release. These results suggest that a signaling cascade comprising ERK and AP-1 activation in IECs is involved in MMP-7 upregulation and syndecan-2 release during exposure to BFT.

## 1. Introduction

Enterotoxin-producing *Bacteroides fragilis* (ETBF) causes several colonic diseases, including inflammation and cancer [1,2]. *B. fragilis* enterotoxin (BFT), a virulence factor of ETBF, is responsible for that pathogenesis [1,3]. BFT secreted by ETBF contacts intestinal epithelial cells (IECs), and the subsequent effects can induce colonic inflammation and breaks in the gut barrier. Maintaining a steady status of epithelial cell-to-cell junctions requires E-cadherin and β-catenin structures in the intestinal barrier [4]. In our previous studies of ETBF infection, we demonstrated that artificially injecting BFT into the murine ileum eventually destroyed gut integrity through villous destruction, neutrophil infiltration, and mucosal congestion [5]. In addition, BFT produced by ETBF cleaves the extracellular domain of E-cadherin that is involved in preserving the integrity of IEC barriers [6]. Matrix metalloproteinase (MMP)-7 is reported to mediate the degradation of E-cadherin in proximal tubular cells, which leads to the nuclear translocation of β-catenin [7]. The nuclear translocation of β-catenin can also control MMP-7 expression in colorectal cancer tissues [8]. We recently reported that BFT induces the translocation of β-catenin into the nuclei of IECs [9]. Based on those observations, we hypothesized that BFT might influence MMP-7 expression. However, little is known about MMP-7 expression in IECs treated with BFT.

MMPs are zinc-dependent proteinases that perform essential functions in controlling the synthesis and degradation of the basement membrane and the extracellular matrix in intestinal barriers [10]. They fulfill their functions by processing non-matrix bioactive substrates associated with membrane shedding, modifying chemokines or growth factors, and modulating the activity of other proteases [10,11]. Therefore, they regulate physiologic functions, including cell proliferation and differentiation, tissue homeostasis, and immunologic responses [10]. MMPs are structurally related but genetically distinct molecules that are classified into five subgroups depending on the structure and specificity of the substrate: (a) collagenases (MMP-1, MMP-8, and MMP-13), (b) gelatinases (MMP-2 and MMP-9), (c) stromelysins (MMP-3, MMP-10, and MMP-11), (d) matrilysins (MMP-7 and MMP-26), and (e) membrane-type (MMP-14, MMP-15, and MMP-16) [8,12]. Our study target, MMP-7, is found constitutively in the IECs and associated with tissue remodeling and the IEC response to infection [13]. In addition, secreted forms of MMP-7 can modify several pathophysiological functions such as tumor metastasis and inflammation [14].

Signals from transcription factors, including nuclear factor-kappaB (NF-κB) and activator protein-1 (AP-1), can control MMP-7 expression [15,16,17,18]. We already demonstrated that stimulating IECs with BFT can activate the signaling of those transcription factors [5,19,20,21,22]. However, there is no evidence that the BFT-induced signaling is associated with MMP-7 induction in IECs. In this study, we explored the regulation of MMP-7 expression in IECs exposed to BFT. We found that signaling pathways comprising ERK mitogen-activated protein kinases (MAPKs) and AP-1 were essential for MMP-7 induction following exposure to BFT. Those results were associated with the shedding of syndecan-2 in BFT-exposed IECs.

## 2. Results

### 2.1. BFT Upregulates MMP-7 Expression in IECs

**Figure 1:**
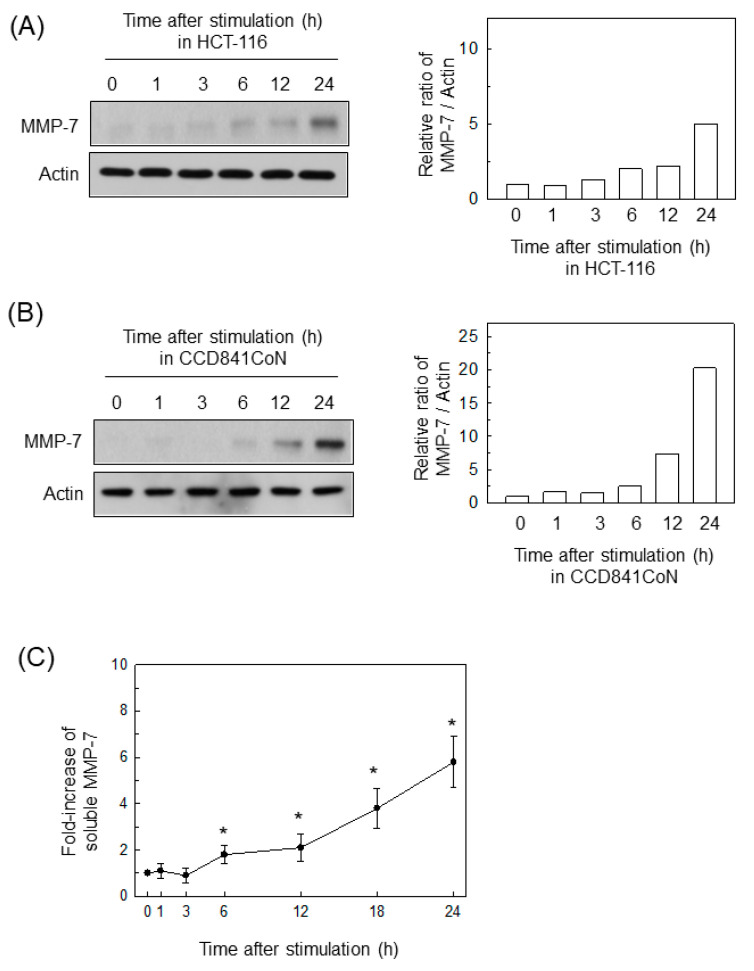
BFT enhances MMP-7 expression in IECs. (**A**,**B**) HCT-116 (**A**) and CCD 841 CoN cells (**B**) were treated with BFT (300 ng/mL) for the indicated periods. Protein expression of pro-MMP-7 and actin was evaluated by Western blotting. All images are representative of more than three independent experiments. Densitometric analysis for expressed proteins represents the relative densities of each protein compared with actin. (**C**) HCT-116 cells were treated with the indicated concentrations of BFT for 24 h. Levels of soluble MMP-7 were analyzed in the conditioned media using an ELISA kit. Values are expressed as the mean ± SEM (*n* = 5). *, *p* < 0.05 compared with the untreated control.

Treating HCT-116 cells with BFT upregulated the expression of MMP-7 proteins (Figure 1A). In addition, CCD 841 CoN cells (a normal colonic epithelial cell line) treated with BFT also increased their MMP-7 expression, as assessed by immunoblotting (Figure 1B). In another experiment, the levels of soluble MMP-7 were measured with an ELISA kit using conditioned medium from HCT-116 cells treated with BFT. As shown in Figure 1C, a significant increase in soluble MMP-7 was first noted 6 h after treatment with BFT and continued to 24 h post-stimulation. 

### 2.2. Activation of NF-κB Is Not Associated with MMP-7 Induction in IECs following BFT Stimulation

**Figure 2:**
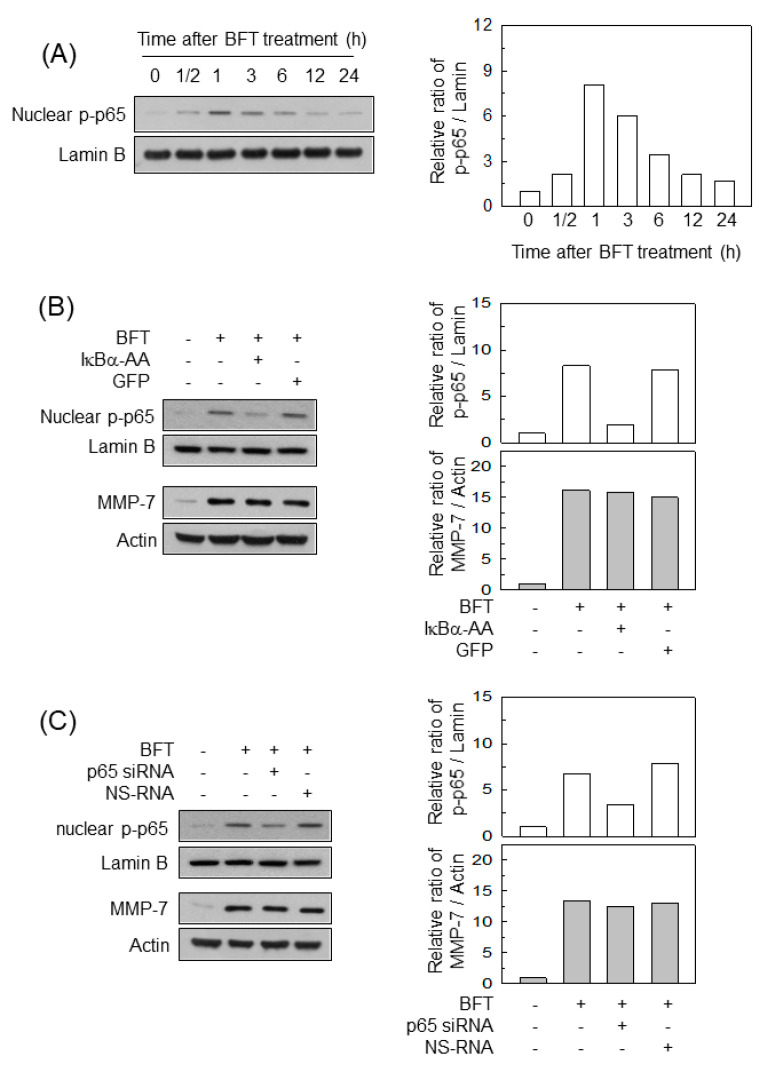
Effects of NF-κB suppression on MMP-7 expression in IECs stimulated with BFT. (**A**) HCT-116 cells were treated with BFT at a concentration of 300 ng/mL for the indicated period. Expression of phospho-p65 and lamin B in nuclear extracts was detected by immunoblotting. (**B**) HCT-116 cells were transfected with a lentivirus containing an IκBα-super-repressor (IκBα-AA) or a control virus (GFP). Transfected cells were stimulated with BFT (300 ng/mL) for 1 h (phos-pho-p65) or 24 h (MMP-7). Expression of phospho-p65 in the nuclear factions and MMP-7 in the whole-cell lysates was assessed by immunoblotting. (**C**) HCT-116 cells were transfected with NF-κB p65-specific siRNA or non-silencing siRNA (NS-RNA) as a control for 48 h, after which the cells were combined with BFT (300 ng/mL) for 1 h (phospho-p65) or 24 h (MMP-7). Expression of phospho-p65 in the nuclear factions and MMP-7 in the whole-cell lysates was assessed by immunoblotting. All results shown are representative of more than three independent experiments. Densitometric analysis for expressed proteins represents the relative densities of each protein compared with actin or lamin B.

The NF-κB transcription factor was activated in BFT-exposed HCT-116 cells (Figure 2A). We next used transfection models to examine whether NF-κB activation was linked to MMP-7 upregulation in IECs. Transfection with lentivirus-IκBα-AA decreased the nuclear phospho-p65 signal to the control level after BFT treatment (Figure 2B, top panels). In this experiment, transfection with lentivirus-IκBα-AA did not significantly change the expression of MMP-7 in HCT-116 cells (Figure 2B, bottom panels). In another experiment, we used p65 siRNA to inhibit NF-κB activity. p65 siRNA suppressed the nuclear protein expression of phospho-p65 in BFT-treated cells (Figure 2C, top panels). However, no change in MMP-7 expression was observed between the cells transfected with p65 siRNA and untransfected cells (Figure 2C, bottom panels). 

### 2.3. AP-1 Is Involved in the Upregulation of MMP-7 in BFT-Stimulated IECs

**Figure 3:**
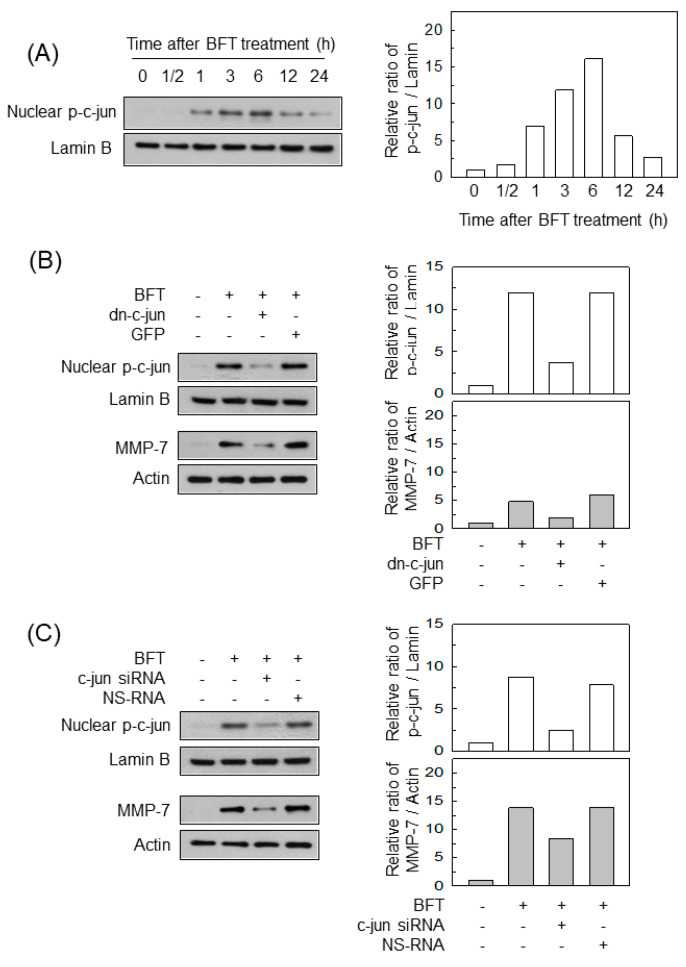
Effects of AP-1 suppression on MMP-7 expression in IECs stimulated with BFT. (**A**) HCT-116 cells were treated with BFT at a concentration of 300 ng/mL for the indicated period. Nuclear protein expression of phospho-c-jun and lamin B was detected by immunoblotting. (**B**) HCT-116 cells were transfected with a lentivirus containing dominant-negative c-jun plasmid (dn-c-jun) or a control virus (GFP). Transfected cells were stimulated with BFT (300 ng/mL) for 1 h (phospho-c-jun) or 24 h (MMP-7). The expression of phospho-c-jun in the nuclear extracts and MMP-7 in the whole-cell lysates was assayed by immunoblotting. (**C**) HCT-116 cells were transfected with c-jun-specific siRNA or non-silencing siRNA (NS-RNA) as a control for 48 h, after which the cells were combined with BFT (300 ng/mL) for 1 h. Expression of phospho-c-jun in the nuclear factions and MMP-7 in the whole-cell lysates was assessed by immunoblotting. All results shown are representative of more than three independent experiments. Densitometric analysis for expressed proteins represents the relative densities of each protein compared with actin or lamin B.

The transcription factor AP-1 was also activated in BFT-exposed HCT-116 cells (Figure 3A). Transfection with lentivirus-dn-c-jun suppressed the phospho-c-jun signal to control levels in BFT-stimulated HCT-116 cells, whereas the control GFP did not diminish (Figure 3B, top panels). In this experiment, transfection with lentivirus-dn-c-jun significantly reduced the levels of MMP-7 expression in HCT-116 cells (Figure 3B, bottom panels). We next used siRNA against c-jun to suppress AP-1 activity. As shown in the top panels of Figure 3C, transfection with siRNA against c-jun inhibited the signals of nuclear phospho-c-jun. In this experiment, c-jun siRNA significantly attenuated MMP-7 expression under BFT-stimulation conditions (Figure 3C, bottom panels).

### 2.4. ERK Is Involved in the Upregulation of MMP-7 in BFT-Stimulated IECs

**Figure 4:**
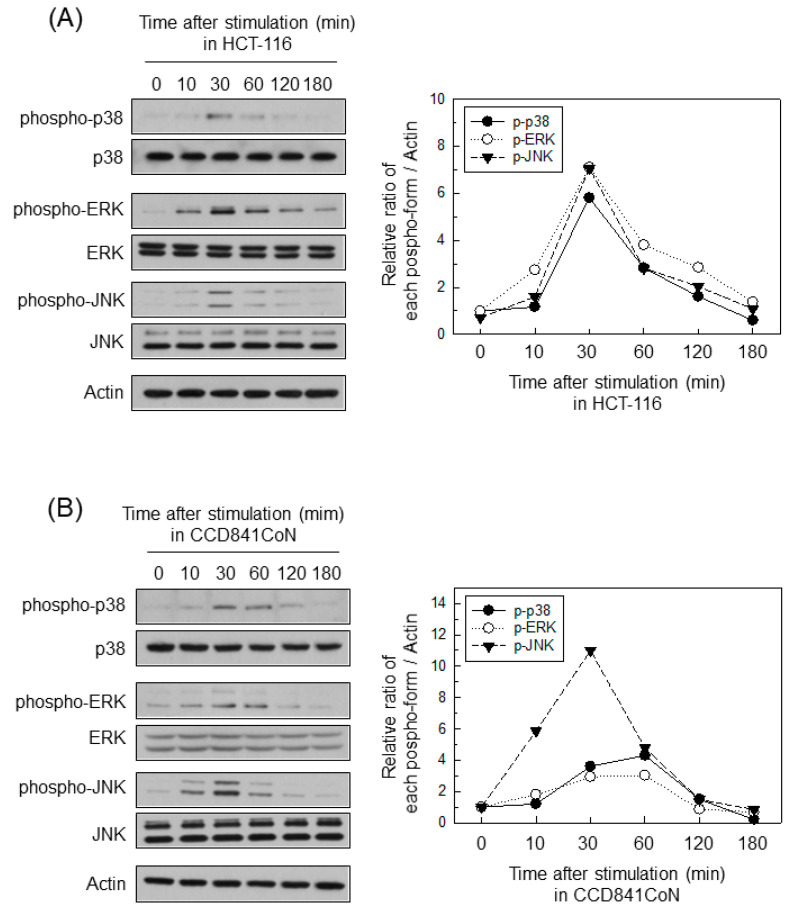

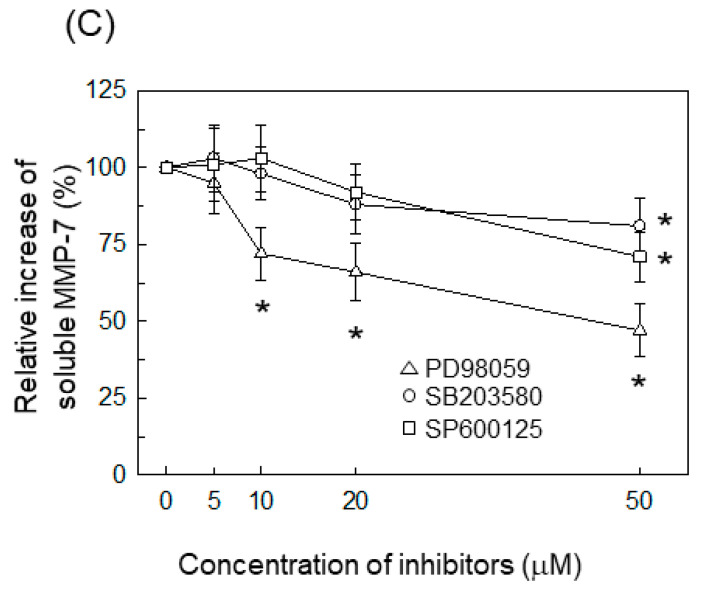
Effects of MAPK chemical inhibitors on MMP-7 expression in IECs stimulated with BFT. (**A**,**B**) CCD 841 CoN (**A**) and HCT-116 cells (**B**) were treated with BFT at a concentration of 300 ng/mL for the indicated period. Protein expression of ERK1/2, phospho-ERK1/2, p38, phospho-p38, JNK and phospho-JNK was detected by immunoblotting. All pictures are representative of more than three independent experiments. Densitometric analysis for expressed proteins of each phospho-form represents the relative densities of each protein compared with actin. (**C**) CCD 841 CoN cells were preincubated with SB203580, SP600125, or PD98059 for 30 min. BFT (300 ng/mL) was then added to each group for 24 h. Levels of soluble MMP-7 were evaluated by ELISA. Data are expressed as a mean % increase relative to unstimulated controls ± SEM (*n* = 5). *, *p* < 0.05 compared with BFT alone.

BFT stimulation activated the phosphorylated forms of MAPK proteins such as ERK1/2, p38, and JNK in HCT-116 cells (Figure 4A). CCD 841 CoN cells treated with BFT also increased their production of the phosphorylated form of each MAPK (Figure 4B). To evaluate the effects of MAPK inhibition on the MMP-7 induction in BFT-treated cells, we used chemical kinase inhibitors as previously described [23,24]. Under BFT-stimulated conditions, MMP-7 expression was first inhibited significantly at 10 μM concentration of PD98059 (ERK inhibitor). In contrast, SB203580 (p38 inhibitor) and SP600125 (JNK inhibitor) first significantly inhibited MMP-7 expression at a concentration of 50 μM (Figure 4C). 

**Figure 5:**
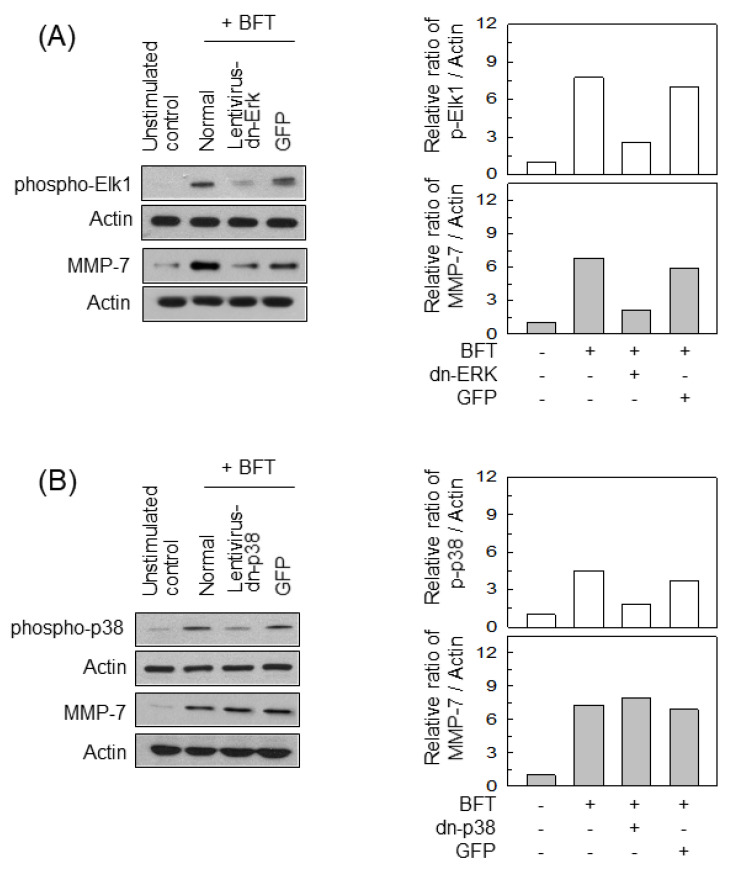

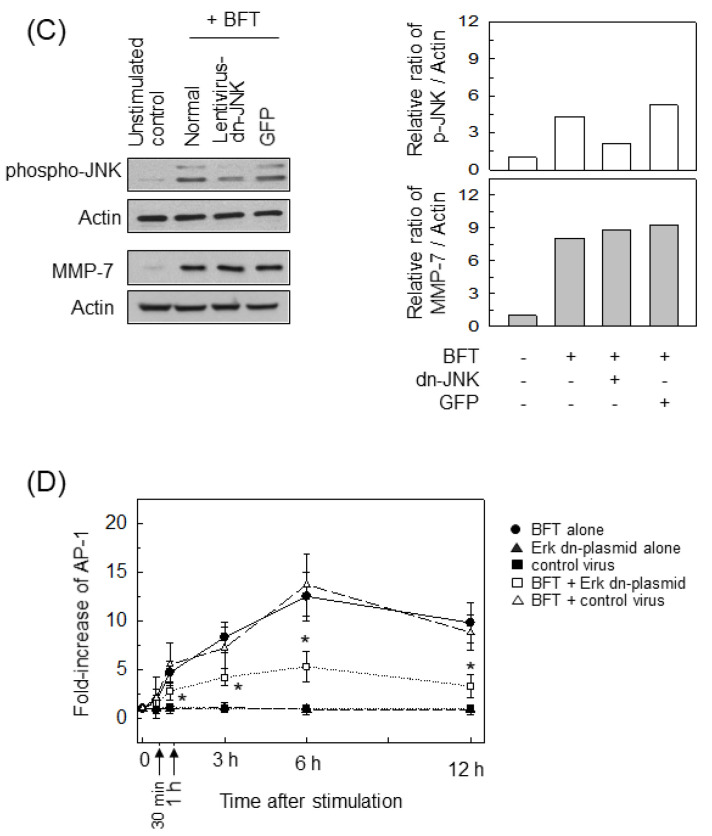
Effects of MAPK suppression on MMP-7 expression in IECs stimulated with BFT. (**A**) HCT-116 cells were transfected with lentiviruses containing a dominant-negative Erk (dn-Erk) or a control plasmid (GFP). Cells were treated with BFT at a concentration of 300 ng/mL for 30 min (top panels, phospho-Elk1) and 24 h (bottom panels, MMP-7). (**B**) HCT-116 cells were transfected with lentiviruses containing a dominant-negative p38 or the control plasmid. The culture conditions were identical to those in (**A**). The top panels show phospho-38, and the bottom panels show MMP-7 signals. (**C**) HCT-116 cells were transfected with lentiviruses containing a dominant-negative JNK or the control plasmid. The culture conditions were identical to those in (**A**). The top panels show phospho-JNK, and the bottom panels show MMP-7. Protein expression was determined by Western blotting. All images in (**A**–**C**) are representative of more than three independent experiments. Densitometric analysis for expressed proteins represents the relative densities of each protein compared with actin or lamin B. (**D**) Each lentivirus-infected cell was treated with BFT (300 ng/mL), and then AP-1 activity was determined by ELISA. Data are expressed as the mean fold induction ± SEM (*n* = 5). *, *p* < 0.05 compared with BFT alone.

We next performed experiments using lentiviral systems containing dominant-negative plasmids to confirm those findings. Transfection with lentiviruses containing a dominant-negative Erk2 plasmid (lentivirus-dn-Erk) suppressed the phosphorylation of Elk1 proteins in HCT-116 cells (Figure 5A, top panels). In this experiment, the lentivirus-dn-Erk significantly decreased MMP-7 expression following BFT stimulation (Figure 5A, bottom panels). In contrast, transfection with lentiviruses containing a dominant-negative p38 plasmid (lentivirus-dn-p38) did not significantly change the expression of MMP-7 in BFT-stimulated HCT-116 cells (Figure 5B). Lentiviral infection with a dominant-negative JNK1 plasmid (lentivirus-dn-JNK) did not affect MMP-7 expression, either (Figure 5C). To further investigate ERK-induced AP-1 activation, we used ELISA kits to measure AP-activity. Infection with lentivirus-dn-Erk reduced AP-1 activity in cells treated with BFT (Figure 5D). Thus, exposing IECs to BFT might trigger a signaling pathway comprising ERK, AP-1, and MMP-7 induction.

### 2.5. BFT-Induced MMP-7 Upregulation Is Associated with Syndecan-2 Release in IECs

**Figure 6:**
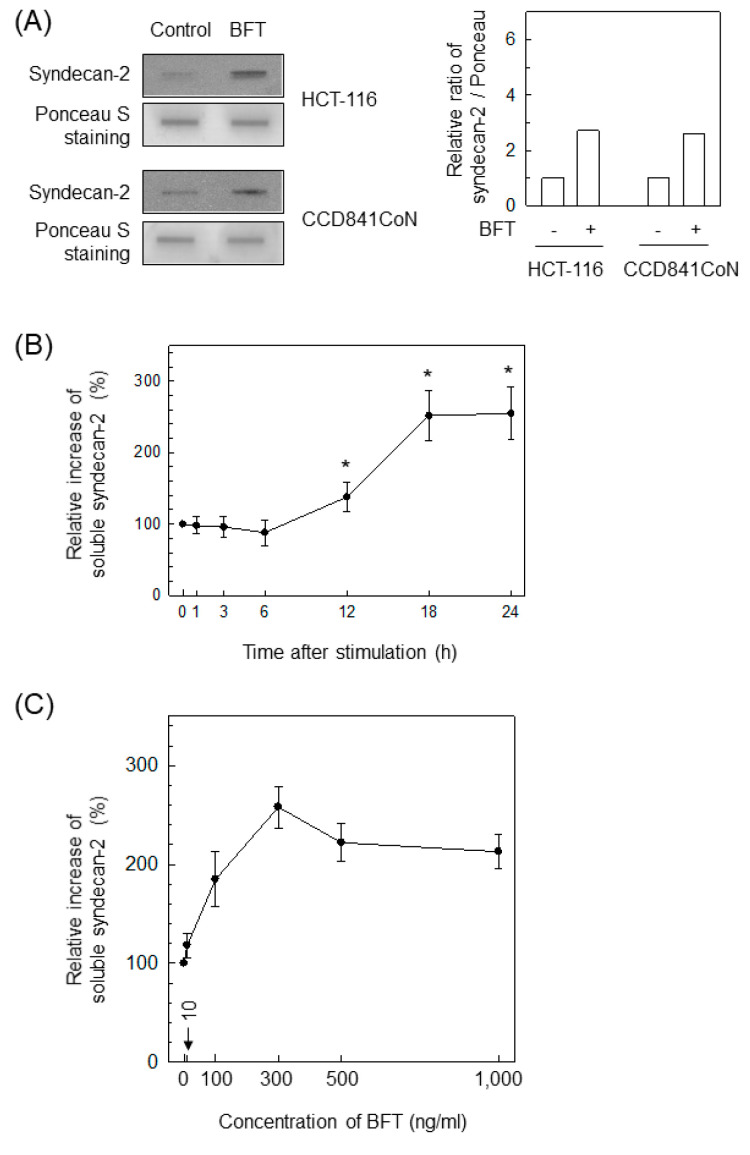
BFT promotes release of syndecan-2 in IECs. (**A**) HCT-116 (top panels) and CCD 841 CoN cells (bottom panels) were treated with BFT (300 ng/mL) for 24 h, and then conditioned media were collected and analyzed by slot blotting with an anti-syndecan-2 antibody. Ponceau S staining was used to check for equal protein loading. All images are representative of more than three independent experiments. Densitometric analysis for expressed proteins represents the relative densities of each protein compared with Ponceau S. (**B**) HCT-116 cells were treated with BFT (300 ng/mL) for the indicated periods. Quantitative analysis of syndecan-2 released in conditioned media was performed using an ELISA kit. * *p* < 0.05 compared with untreated control. (**C**) HCT-116 cells were treated with the indicated concentrations of BFT for 24 h. Levels of soluble syndecan-2 were analyzed in the conditioned media by an ELISA kit. Values are expressed as the mean ± SEM (*n* = 5). *, *p* < 0.05 compared with the untreated control.

Because the extracellular domain of syndecan-2 is cleaved by MMP-7 in colon cancer cells [16,25], it is likely that MMP-7 is involved in syndecan-2 shedding when BFT contacts IECs. To investigate that hypothesis, we first examined whether BFT could induce syndecan-2 release in IECs. As shown in the top panels of Figure 6A, treating HCT-116 cells with BFT enhanced the levels of soluble syndecan-2. CCD 841 CoN cells exposed to BFT also increased soluble syndecan-2 levels in slot blot and conditioned culture supernatant assessments (Figure 6A, bottom panel). In the following experiment, the concentration of soluble syndecan-2 was measured using an ELISA kit with conditioned culture supernatants from HCT-116 cells treated with BFT. As shown in Figure 6B, a significant increase in soluble syndecan-2 was first noted 12 h following BFT exposure and continued to 24 h post-stimulation. The increase in soluble syndecan-2 depended on the concentration of BFT used for stimulation (Figure 6C). 

**Figure 7:**
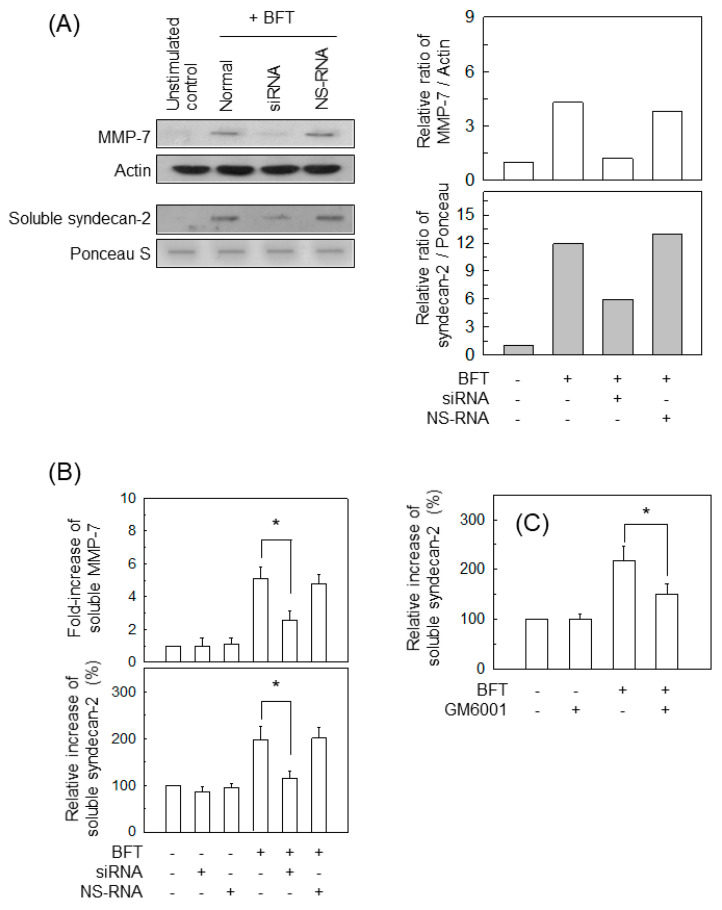
Effects of MMP-7 suppression on syndecan-2 release in IECs stimulated with BFT. (**A**) HCT-116 cells were transfected with either MMP-7 siRNA or non-silencing siRNA (NS-RNA). Cells were treated with BFT at a concentration of 300 ng/mL for 24 h. Protein expression of MMP-7 and actin was detected by Western blotting (top panels). The soluble form of syndecan-2 was evaluated using conditioned media and slot blotting with each antibody. Protein loading in blots was determined by Ponceau S staining (bottom panels). All images are representative of more than three independent experiments. Densitometric analysis for expressed proteins represents the relative densities of each protein compared with actin or Ponceau S. (**B**) HCT-116 cells were treated with the indicated concentrations of BFT for 24 h. Levels of soluble MMP-7 and syndecan-2 in the conditioned media were analyzed using ELISA kits. Values are expressed as the mean ± SEM (*n* = 5). (**C**) CCD 841 CoN cells were treated with BFT (300 ng/mL) in the presence and absence of GM6001 (10 μM) for 24 h. Levels of soluble syndecan-2 were analyzed in the conditioned media using ELISA kits. Values are expressed as the mean ± SEM (*n* = 5). *, *p* < 0.05 compared with the untreated control.

We next examined whether BFT-induced MMP-7 upregulation is related to syndecan-2 release in IECs. A transfection model with siRNA was used to suppress MMP-7 signals in BFT-exposed cells. The experiment using whole-cell lysate obtained from MMP-7 siRNA-transfected cells showed the apparent suppression of MMP-7 signals under BFT-stimulated conditions (Figure 7A, top panels). As assessed by slot blotting and the conditioned culture supernatant, MMP-7 siRNA transfection led to a significant reduction in soluble syndecan-2 compared with untransfected cells stimulated with BFT, and transfection with NS-RNA as a control had no significant effect (Figure 7A, bottom panels). In another experiment, BFT significantly increased both soluble MMP-7 and soluble syndecan-2 in the conditioned media. In that experiment, transfection with MMP-7 siRNA significantly decreased the levels of soluble MMP-7 and soluble syndecan-2 (Figure 7B). In the next experiment, CCD 841 CoN cells were preincubated with GM6001 (10 μM), a broad-spectrum MMP inhibitor, and then treated with BFT. As shown in Figure 7C, GM6001 significantly suppressed syndecan-2 release compared with untreated cells. Those results might allow the establishment of a theory of MMP-7-dependent induction of syndecan-2 release in IECs exposed to BFT.

### 2.6. AP-1 Signaling Is Involved in Syndecan-2 Release in IECs Stimulated with BFT

**Figure 8:**
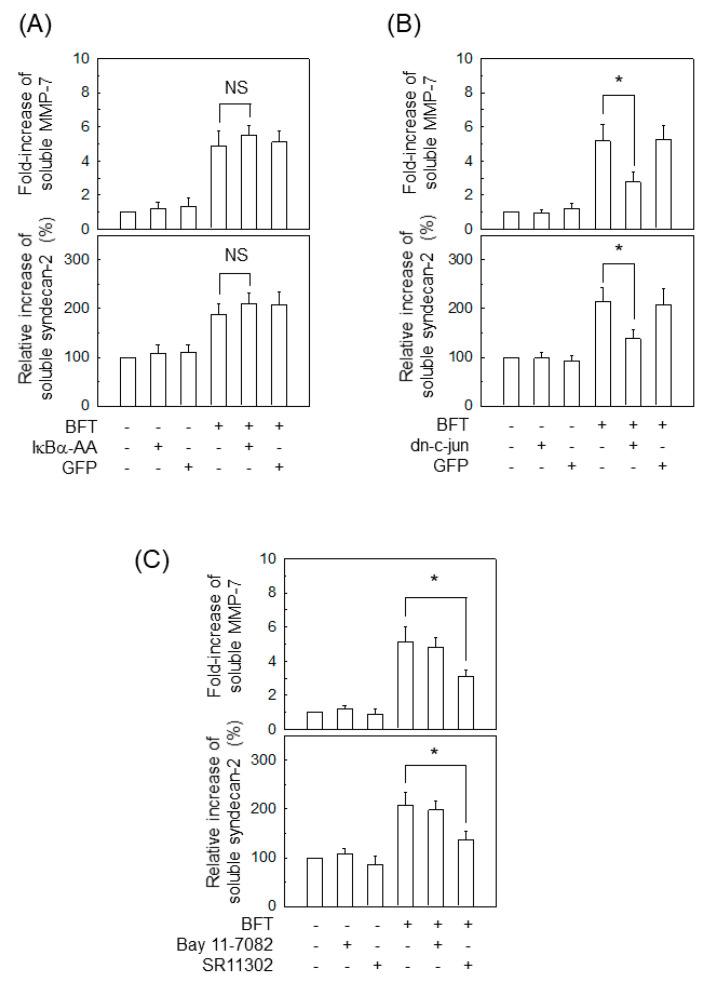
Effects of transcription factor suppression on syndecan-2 shedding in IECs stimulated with BFT. (**A**) The culture conditions for the HCT-116 cells were identical to those in Figure 5A–C using those respective lentiviral vectors. Cells were treated with BFT at a concentration of 300 ng/mL for 24 h. Levels of soluble MMP-7 and syndecan-2 in the conditioned media were analyzed using an ELISA kit. Values are expressed as the mean ± SEM (*n* = 5). (**B**) HCT-116 cells were transfected with dn-c-jun or GFP, as described in Figure 3B. Cells were treated with BFT at a concentration of 300 ng/mL for 24 h. Levels of soluble MMP-7 and syndecan-2 in the conditioned media were analyzed using an ELISA kit. Values are expressed as the mean ± SEM (*n* = 5). * *p* < 0.05 compared with the untreated control. (**C**) CCD 841 CoN cells were preincubated with Bay 11-7082 (50 μM) or SR11302 (10 μM) for 30 min, followed by stimulation with BFT (300 ng/mL) for an additional 24 h. Levels of soluble MMP-7 and syndecan-2 in conditioned media were measured using ELISA (mean ± SEM, *n* = 5). * *p* < 0.05 compared with BFT alone. NS, statistically non-significant.

We used a lentivirus-transfection system to examine the involvement of transcriptional factors in syndecan-2 shedding. In the experiment that used lentivirus-IκBα-AA transfection (Figure 2B), suppressing NF-κB activity did not affect soluble MMP-7 or syndecan-2 upon BFT exposure (Figure 8A). In contrast, transfection with lentivirus-dn-c-jun significantly reduced the levels of soluble MMP-7 and syndecan-2 in HCT-116 cells (Figure 8B). Primary intestinal epithelial CCD 841 CoN cells were pre-incubated with Bay 11-7082 (NF-κB inhibitor) or SR11302 (AP-1 inhibitor) for 30 min, and then stimulated with BFT to confirm those results. As shown in Figure 8C, pretreating CCD 841 CoN cells with SR11302 resulted in a significant decrease in soluble MMP-7 and soluble syndecan-2 compared with treatment with BFT alone. However, pretreatment with Bay 11-7082 did not significantly change the BFT-induced levels of soluble MMP-7 and syndecan-2. 

### 2.7. MMP-7-Associated ERK Activation Is Essential for Syndecan-2 Release in BFT-Stimulated IECs

**Figure 9:**
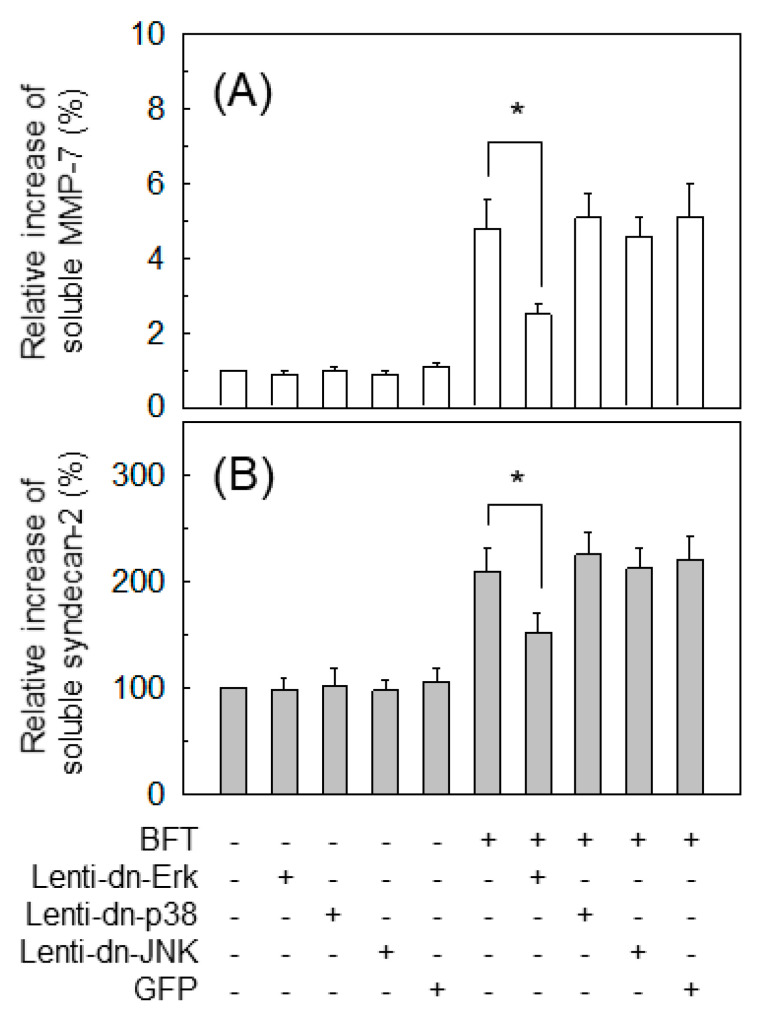
Effects of MAPK suppression and syndecan-2 shedding in IECs Scheme 116. cells were identical to those in Figure 5A–C using those respective lentiviral vectors. Cells were treated with BFT at a concentration of 300 ng/mL for 24 h. Levels of soluble MMP-7 (**A**) and syndecan-2 (**B**) were determined using ELISA kits. Data are expressed as the mean % increase relative to unstimulated controls ± SEM (*n* = 5). * *p* < 0.05 compared with BFT alone.

In the previous experiments, BFT induced the phosphorylated forms of MAPK proteins such as p38, ERK, and JNK in HCT-116 cells (Figure 4A,B). We used lentiviral systems containing dominant-negative plasmids (Figure 5A–C) to assess the effects of MAPK signals on syndecan-2 release. Lentiviral infection with dn-Erk significantly decreased MMP-7 expression compared with the untransfected control (Figure 9A). But lentiviral infection with dn-p38 or dn-JNK produced no changes in MMP-7 expression under BFT-stimulated conditions. Similar results were obtained for soluble syndecan-2 levels in cells transfected with dn-Erk under BFT-stimulated conditions (Figure 9B). 

## 3. Discussion

IECs exposed to BFT can express mediators, such as IL-8 and β-catenin, and transcription factors, such as AP-1 and NF-κB, to regulate the expression of those effector molecules [5,9,19,20,21,22]. Concurrently, BFT can affect several components needed to maintain the IE barrier. In this study, we showed that BFT upregulates the expression of MMP-7 and that the enhanced MMP-7 expression is associated with syndecan-2 release in IECs stimulated with BFT.

Many MMPs are synthesized and then secreted as proenzymes; MMP-7 is a soluble-type MMP [26,27] that can be upregulated in IECs [28]. MMP-7 degrades a variety of matrix substrates such as elastin, gelatin, and proteoglycans. Because secreted MMP-7 might play a role in the pathogenesis of early-stage colon tumors [28], we measured its form using an ELISA kit and conditioned medium from BFT-treated cells. The cellular forms of MMP-7 were also measured using Western blotting and cell lysates in this study. Our results show that BFT can increase both the cellular and secreted forms of MMP-7 in IECs.

Which transcription factor is responsible for MMP-7 induction is controversial. For example, isoproterenol might induce AP-1-mediated MMP-7 expression in gastric cancer cells [18], and hydrogen peroxide caused the expression of MMP-7 molecules in SW-620 human colon cancer cells via an AP-1 signaling pathway [15]. In contrast, IL-1α- or TNF-α-treated IECs increased their expression of MMP-7 proteins via an NF-κB activation pathway [16,17]. In this study, exposing HCT-116 cells to BFT increased MMP-7 protein expression, and BFT-activated AP-1 signaling was involved in MMP-7 upregulation. Based on those findings, we investigated the upstream signaling associated with BFT-induced MMP-7 upregulation. 

MAPK signaling is known to be an essential piece underlying the expression of several target proteins, including MMP-7 and syndecan-2. Several reports have demonstrated MAPK signaling-associated MMP-7 expression. For example, animal experiments showed that treating mice with JNK- or ERK-specific inhibitors decreased MMP-7 expression in tumor tissue, suggesting that MMP-7 induction occurs via the activation of JNK and ERK [29]. In addition, human peritoneal mesothelial cells express MMP-7 molecules via ERK activation [30]. Stimulating HT-29 cells with IL-1α enhanced their MMP-7 protein secretion via activation of phospho-ERK and phospho-p38 MAPK molecules [16]. In this study, the suppression of ERK molecules suppressed both the AP-1 signal and MMP-7 expression in primary intestinal epithelial CCD 841 CoN and HCT-116 cells treated with BFT. In contrast, inhibiting p38 or JNK activity did not influence the MMP-7 expression in IECs stimulated with BFT. Therefore, the mechanisms of ERK-associated MMP-7 induction and AP-1 activation in IECs might be stimulator-specific and seem to depend on the type of IECs.

A previous study reported that BFT could not activate the MMP-7 signaling pathway because no active form of the MMP-7 molecules was observed in Western blot assays of control or BFT-stimulated HT29/C1 cells [31]. Nevertheless, we found that MMP-7 molecules, one of the constitutive components of IECs, were upregulated in IECs exposed to BFT and that enhanced MMP-7 expression was related to syndecan-2 release. HT-29 cell lines constitutively express high levels of MMP-7 proteins in the steady state. In contrast, HCT-116 cell lines express a relatively low level of MMP-7 molecules in their stable condition [4]. Based on that finding, we used HCT-116 cell lines in this study. Nevertheless, we did not experiment with immunofluorescence experiment to confirm the status of MMP-7. It seems necessary to experiment on this issue.

Syndecans are the dominant forms of surface heparan sulfate proteoglycans in eukaryotic cells. Among microbial infections, *Brucella melitensis*, *Pseudomonas aeruginosa, Neisseria gonorrhoeae*, and other bacteria such as *Staphylococcus* and *Streptococcus* species attach and invade hosts by acting together with syndecan molecules [32,33,34,35]. Syndecan-2 is known to be involved in a variety of functions such as cell proliferation, migration, and interaction between cells and intercellular substances as well as microbial interaction. For example, syndecan-2 expressed on the surface of dendritic cells binds to HIV, after which syndecan-2 facilitates viral transmission to CD4-positive T cells [36]. The syndecan-2 knock-out state diminished both phagocytic activity against apoptotic neutrophils and the ability to convert macrophages from a proinflammatory phenotype to a pro-resolution one in mesenchymal stromal cells [37]. *Helicobacter pylori* infection also increased the soluble level of syndecan-2 released from epithelial cells [28]. However, nothing has been reported about the role of syndecan-2 in ETBF infection until the results of this study, which is the first report to elucidate the role of syndecan-2 in ETBF infection.

We found that PD98059 (ERK inhibitor) was superior to both SB203580 and SP600125 in diminishing MMP-7 upregulation and syndecan-2 release. We confirmed those findings by using a lentivirus-based knockdown strategy. Considering that the extracellular domain of shed syndecan-2 plays an essential role in the pro-MMP-7 activation process in IECs [38], the syndecan-2 release induced by BFT could be involved in the pro-MMP-7 activation process in IECs. Further exploration is needed to clarify the roles played by the syndecan-2 released in ETBF infection. 

Based on the present findings, we hypothesize that BFT activates a signaling cascade comprising ERK and AP-1 activation that is related to MMP-7 upregulation and syndecan-2 release in IECs. Our proposal could be a fruitful avenue for future investigation of ETBF infection. Nevertheless, this study has several limitations. We applied a pharmacological dose of BFT to promote MMP-7 upregulation and the syndecan-2 release. In addition, we did not examine whether the released syndecan-2 controlled MMP-7 upregulation or the pro-MMP-7 activation process in IECs stimulated with BFT. Future exploration is needed to clarify whether ETBF infection affects MMP-7 expression and syndecan-2 release in vivo and whether the released syndecan-2 regulates the pro-MMP-7 activation process in BFT-exposed IECs. We did not perform cell migration analysis to evaluate the effect of MMP-7 production. Therefore, it is necessary for experiments to study whether MMP7 can promote cell migration using wound-healing assays and Transwell migration and invasion assays. In conclusion, MMP-7 upregulation in BFT-exposed IECs was closely related to syndecan-2 release via the BFT-induced activation of AP-1 and ERK signals. 

## 4. Materials and Methods

### 4.1. Reagents

We used the following reagents in this study: antibiotics (mixture of 100 μg/mL of streptomycin and 100 units/mL of penicillin), Trizol, Ca^2+^- and Mg^2+^-free Hank’s balanced salt solution (GIBCO BRL, Gaithersburg, MD, USA); Eagle’s minimum essential medium (EMEM), McCoy’s 5a medium, and fetal bovine serum (FBS) (American Type Culture Collection (ATCC), Manassas, VA, USA); Bay 11-7085, SB203580, PD98059, and SP600125 (Calbiochem, La Jolla, CA, USA); GM6001 and Ponceau S (Sigma-Aldrich, St. Louis, MO, USA); and SR11302 (MedChemExpress, Monmouth Junction, NJ, USA).

The following antibodies were used in this study: rabbit monoclonal antibodies (mAbs) against phospho-IκBα and rabbit polyclonal Abs against phospho-p65, phospho-c-jun, pan-extracellular signal-regulated kinase 1/2 (ERK1/2, p44/p42), phospho-ERK1/2, pan-p38, phospho-p38, pan-JNK (p54/p46), and phospho-JNK (Cell Signaling Technology, Inc., Beverly, MA, USA); mouse mAb against human MMP-7 (R&D Systems, Minneapolis, MN, USA); rabbit polyclonal Ab against human syndecan-2 (Thermo Fisher Scientific, Waltham, MA, USA); and mouse mAbs against actin and lamin B, goat anti-mouse, and anti-rabbit secondary Abs conjugated to horseradish peroxidase (Santa Cruz Biotechnology, Inc., Santa Cruz, CA, USA).

### 4.2. Cell Culture Conditions and Purification of BFT

Cell culture conditions were identical to those reported in our previous studies [9,22,39]. The human colonic epithelial HCT-116 cell line (ATCC CCL 247) was cultured in McCoy’s 5a medium complemented with 10% FBS and antibiotics. Normal colonic epithelial CCD 841 CoN cells (ATCC CRL-1790) were cultured with EMEM with 10% FBS and 1 mM sodium pyruvate [9,22]. BFT was purified from culture supernatants of a toxigenic *B.* *fragilis* strain (ATCC 43858) using protocols detailed in our previous reports [9,19,20,21,22,39].

### 4.3. Transfection Assay

Lentiviral systems containing mammalian expression vectors were used to block NF-κB, AP-1, or MAPK activation, as described previously [9,21,22]. Lentiviral vectors and effective viruses were supported by BioCore at the Institute of Biomedical Science (Seoul, Korea). Experiments related to transfection were performed according to the relevant manufacturer instructions [9,21,22]. 

In this study, we used small interfering RNA (siRNA) against MMP-7, NF-κB p65, c-jun and a non-silencing siRNA (NS-RNA) as the negative control (Santa Cruz Biotechnology, Inc.). Transfected cells were incubated for 48 h before BFT treatment. Transfection experiments were performed according to a protocol described previously [9,21,22]. 

### 4.4. Immunoblots and ELISA

Expressed proteins were detected using immunoblot analyses as described previously [9,21,22]. An amount of 15 to 50 µg of protein per lane were size-fractionated on a polyacrylamide minigel (Mini-PROTEIN II, Bio-Rad) and transferred to a nitrocellulose membrane (0.1-μm pore size; MilliporeSigma, Burlington, MA, USA) via electrophoretic transfer. Secondary Abs conjugated to horseradish peroxidase were reacted with the target proteins bound with the primary Ab. The reaction signals were detected using a West-Q Chemiluminescent Substrate Kit Plus (GenDEPOT, Katy, TX, USA) and X-ray film exposure. 

For slot blotting, the culture supernatants were slot-blotted to a PVDF membrane (MilliporeSigma) using a Bio-Dot SF microfiltration apparatus (Bio-Rad, Hercules, CA, USA). Equal amounts of protein were loaded onto the slot blots. Before blotting, the membrane was stained with Ponceau S. The slot blotting and immunoreaction with the anti-syndecan-2 Ab were performed according to a previously described method [16]. The reaction signals were detected using a West-Q Chemiluminescent Substrate Kit Plus (GenDEPOT) and X-ray film exposure.

A p44/42 MAP kinase assay kit (Cell Signaling Technology) was used to measure phospho-Elk1 molecules as described previously [9,21,22]. An ELISA kit for Trans^AM®^ AP-1 was obtained from Active Motif (Carlsbad, CA, USA). Equal volumes of conditioned media were obtained from cells and the protein levels in the culture supernatants following BFT stimulation were measured using commercially available ELISA kits for soluble MMP-7 (Invitrogen, Carlsbad, CA, USA) and soluble syndecan-2 (LifeSpan Biosciences Inc., Seattle, WA, USA). Each assay was performed according to the relevant manufacturer instructions.

### 4.5. Statistical Analyses

Data are indicated as the mean ± SEM and the Mann–Whitney t-test was used for the statistical analyses. A *p* value less than 0.05 was considered statistically significant.
